# A long-term prospecting study on giant viruses in terrestrial and marine Brazilian biomes

**DOI:** 10.1186/s12985-024-02404-z

**Published:** 2024-06-10

**Authors:** Talita B. Machado, Isabella L. M. de Aquino, Bruna L. Azevedo, Mateus S. Serafim, Matheus G. Barcelos, Ana Cláudia S. P. Andrade, Erik Reis, Leila Sabrina Ullmann, João Pessoa, Adriana O. Costa, Luiz H. Rosa, Jônatas S. Abrahão

**Affiliations:** 1 Laboratório de vírus, Departamento de Microbiologia, Instituto de Ciências Biológicas, Universidade Federal de Minas Gerais (UFMG), Belo Horizonte city, Minas Gerais Brazil; 2 Centre de Recherche du Centre Hospitalier Universitaire de Québec, Université Laval, Laval city, Québec Canada; 3 Laboratório de virologia básica e aplicada, Departamento de Microbiologia, Instituto de Ciências Biológicas, Universidade Federal de Minas Gerais (UFMG), Belo Horizonte city, Minas Gerais Brazil; 4 Laboratório de Virologia Veterinária, Faculdade de Medicina Veterinária e Zootecnia (FAMEZ), Universidade Federal de Mato Grosso do Sul (UFMS), Campo Grande city, Mato Grosso do Sul Brazil; 5 Laboratório de Virologia, Departamento de Microbiologia e Imunologia, Instituto de Biotecnologia, Universidade Estadual Paulista (UNESP), Botucatu city, São Paulo, Brazil; 6 Departamento de Análises Clínicas e Toxicológicas, Faculdade de Farmácia, Universidade Federal de Minas Gerais (UFMG), Belo Horizonte city, Minas Gerais Brazil; 7 Laboratório de Microbiologia Polar e Conexões Tropicais, Departamento de Microbiologia, Instituto de Ciências Biológicas, Universidade Federal de Minas Gerais (UFMG), Belo Horizonte city, Minas Gerais Brazil

**Keywords:** Giant virus, Amoebas, Biomes, Diversity, Richness

## Abstract

The discovery of mimivirus in 2003 prompted the search for novel giant viruses worldwide. Despite increasing interest, the diversity and distribution of giant viruses is barely known. Here, we present data from a 2012–2022 study aimed at prospecting for amoebal viruses in water, soil, mud, and sewage samples across Brazilian biomes, using *Acanthamoeba castellanii* for isolation. A total of 881 aliquots from 187 samples covering terrestrial and marine Brazilian biomes were processed. Electron microscopy and PCR were used to identify the obtained isolates. Sixty-seven amoebal viruses were isolated, including mimiviruses, marseilleviruses, pandoraviruses, cedratviruses, and yaraviruses. Viruses were isolated from all tested sample types and almost all biomes. In comparison to other similar studies, our work isolated a substantial number of Marseillevirus and cedratvirus representatives. Taken together, our results used a combination of isolation techniques with microscopy, PCR, and sequencing and put highlight on richness of giant virus present in different terrestrial and marine Brazilian biomes.

**Supplementary Information:**

The online version contains supplementary material available at 10.1186/s12985-024-02404-z.

## Introduction

Amoeba giant viruses are well-known for their structural and genomic complexity. Since the mimivirus discovery in 2003 [1], several groups of giant viruses have been described. Metagenomics and prospective studies involving virus isolation revealed that giant amoeba viruses are distributed in a diverse range of environments and substrates [2–8]. These entities have been already isolated from water samples, animals’ bodies, permafrost, ocean depths, thermal springs, and soda lakes [9–12].

Brazil is one of the countries with the highest biodiversity and species richness in the world [13]. Its tropical forests, such as the Amazon, are globally renowned for their complexity and their role in regulating the world’s climate. However, tropical forests represent just a part of the vast Brazilian territory. Brazil is described as having six major biomes: Amazon (i) and Atlantic Forest (ii), both typical of humid tropical forests and biodiversity hotspots; Cerrado (iii), a savanna biome with a rich network of rivers and a high number of endemic species; Caatinga (iv), an arid biome with species well-adapted to water scarcity; Pantanal (v), one of the world’s largest wetland plains; and Pampas (vi), a biome typical of the cooler regions of Brazil, composed of grasslands [14]. In this context, our group has been prospecting viruses of amoebas in Brazilian territory, and we have described completely new species, including Tupanvirus and Yaravirus [11, 15].

In this present work, we describe our efforts in the prospective study of giant viruses from 2012 to 2022. A total of 67 viruses were isolated and identified, from almost all Brazilian biomes. Our study provides information into the isolation and richness of giant amoeba viruses across Brazilian territory, shedding light on their presence in both natural and urban environments.

## Materials and methods

### Cells and medium

The free-living amoeba of the species *Acanthamoeba castellanii* from the American Type Culture Collection (ATCC 30,234; Maryland, USA) was used in all experiments. The amoebas were propagated in cell culture flasks using peptone-yeast extract-glucose medium (PYG) supplemented with 100 IU/mL of penicillin (Cellofarm, Brazil), 0.25 µg/mL of amphotericin B (Cultilab, Brazil), and 0.1 mg/mL of streptomycin (Sigma-Aldrich, USA). To perform the subcultures, the amoebas were mechanically detached from the monolayer by tapping the bottle, quantified in a Neubauer chamber, and the necessary amount was inoculated into a new flask with fresh medium.

### Samples collection

**Fig. 1:**
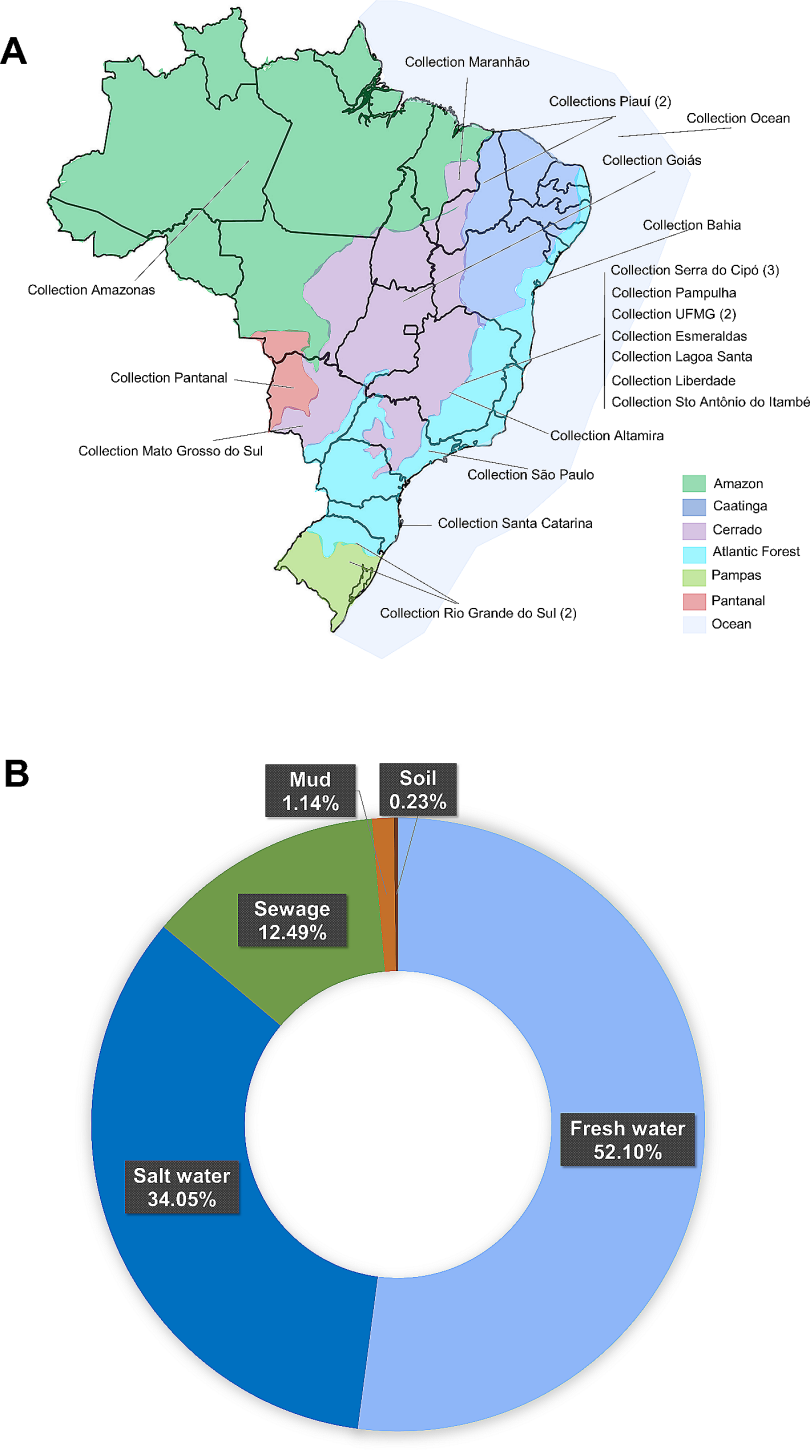
(**A**) Map with the distribution of collections. Location of each collection based on its geographical coordinates. Brazilian biomes are represented by different colors, as per the figure legend. (**B**) Types of samples considering all the collections

The samples were collected from 2012 to 2022. It is important to note that this study does not intend to test a similar number of samples per biome, state, or substrate. This is a descriptive work on the richness of giant viruses found in Brazil in a 11-years study. Brazil has a vast territory, and reaching some regions can be challenging due to logistical or funding issues. Therefore, several collections prospected here were donated to our group by local researchers. The collections were carried out with authorization from the Biodiversity Authorization and Information System (SISBIO) under the identification numbers: 33326-2 and 34293-2; and SISGEN: A2291C9. Most of the collections were made using mainly sterile 1.5 mL microtubes or 50 mL conical tubes. After collection, the samples underwent three cycles of freezing and thawing (a process to reduce contamination) and were organized into collections. For this purpose, each sample was aliquoted into 1.5 mL microtubes, with 1 to 10 aliquots of 1 mL, depending on the initial quantity of the sample, and stored at -20 °C. At the end of the organization, 24 collections were obtained, collected from different Brazilian states and biomes (Fig. 1A). Samples were obtained from different Brazilian states: Amazonas, Bahia, Goiás, Maranhão, Mato Grosso do Sul, Minas Gerais, Piauí, Rio Grande do Sul, Santa Catarina, and São Paulo. These samples resulted in 1 collection from the Pantanal biome, 1 collection from the Caatinga biome, 1 collection from the Amazon biome, 1 collection from the Pampa biome, 6 collections from the Atlantic Forest biome, 13 collections from the Cerrado biome (Sup. Table 1). At last, 61 samples were also collected from the Atlantic Ocean inside the Brazilian Exclusive Economic Zone (EEZ) from Rio de Janeiro to Rio Grande do Norte, corresponding to 1 collection. From those 24 collections, a total of 881 aliquots of 187 samples were tested for giant viruses. The majority of the samples originated from freshwater (459 aliquots), followed by sewage (110 aliquots), saltwater (300 aliquots), mud (10 aliquots), and soil (2 aliquots) (Fig. 1B).

### Prospection of giant viruses in *Acanthamoeba castellanii*

For prospection of giant viruses, preliminary processing of the samples is carried out. For water and sewage samples, a dilution was performed at a ratio of 1:10 for each aliquot. For mud samples, it was necessary to wait 24 h at 10 °C for the sediment to settle first and then perform a dilution at a ratio of 1:10 for each aliquot with the liquid content of the supernatant. For soil samples, 100 g of sediment was collected and 300 µL of sterile distilled water was added, resulting in an initial dilution of 1:4; this content was then vortexed, and the sample was left in the refrigerator overnight for sediment settling. After this period, a dilution at a ratio of 1:10 for each aliquot was performed with the liquid content of the supernatant. The 1:10 dilutions were made using 1x phosphate-buffered saline (PBS). Virus isolation was performed in *Acanthamoeba castellanii* seeded in 96-well plates (Kasvi, Brazil) is PYG medium supplemented with three additional antibiotics: 0.004 mg/mL vancomycin (Inlab, Brazil), 0.004 mg/mL ciprofloxacin (Sigma-Aldrich, USA), and 0.020 mg/mL doxycycline (Sigma-Aldrich, USA). Inoculated amoebas were monitored for seven days, during three rounds of blind passages. In case of cytopathic effects, the extract of amoebas was collected and sent to virus identification [16].

### Transmission electron microscopy

For transmission electron microscopy (TEM), 1.5 × 10^7^ cells of *A. castellanii* were added to T-75 cell culture flasks (Kasvi, Brazil). The cells were infected with the newly isolated viruses during this work at a multiplicity of infection (MOI) of 0.01 and maintained in incubators at a temperature of approximately 30 °C until the cytopathic effect appearance. After observing the cytopathic effect, the contents of the bottle were collected into a 50 mL conical tube and centrifuged at 1308 x g (Sorvall RT6000B) for 10 min. Following centrifugation, the supernatant was discarded, and the pellet was washed with 5 mL of 0.1 M monosodium phosphate buffer, and the contents were transferred to a 15 mL conical tube and centrifuged again at 1308 x g (Sorvall RT6000B) for 10 min, washing and centrifugation were performed twice. After washing, the supernatant was discarded, and the pellet was resuspended in 1.5 mL of fixative composed of 2.5% glutaraldehyde and 0.1 M monosodium phosphate buffer and kept in a homogenizer for 2 h at room temperature. After the incubation period with the fixative, the contents of the conical tube were centrifuged at 145 x g (Sorvall RT6000B) for 10 min, the supernatant was discarded, and the pellet was resuspended with 1 mL of 0.1 M monosodium phosphate buffer, the contents were transferred to a 1.5 mL microtube and centrifuged again at 0.8 x g (Eppendorf 5415R) for 10 min. After this final centrifugation, the material was correctly identified and stored in the refrigerator until it was sent to the Microscopy Center of UFMG. There, it underwent subsequent fixation with 2% osmium tetroxide, embedding in EPON resin, and preparation of ultra-thin sections. Image analysis was performed using a transmission electron microscope (FEI SpiritBiotwin 120 kV). The identification of the viruses was performed based on multiple features from the particles (morphology, size, unique structures and capsid characteristics) combined with impact of infection on the host (viral factory appearance and reorganization of host cytoplasm).

### PCR identification and sequencing

Following the identification of cultures exhibiting a cytopathic effect, screening was conducted via PCR targeting specific giant virus groups (Sup. Table 2). DNA extraction was performed using the phenol-chloroform method from 200 µL of the collected content from positive aliquots, yielding DNA at a concentration of approximately 50 µg/µl, utilized as a template for PCR assays. PCR assays targeted various genes, including the major capsid protein gene of mimivirus, Marseillevirus and yaravirus; and the DNA polymerase gene of Pandoravirus, pithovirus and cedratvirus. Design and standardization of primers and reactions were ensured to prevent cross-amplification among analyzed viruses available on GenBank.

PCR assays utilized 1 µL of extracted DNA (~ 50 nanograms) in an amplification reaction mix containing 5 µL of SYBR Green Master Mix (Thermo Fisher Scientific, USA) and 0.4 µL (10 µM) of forward and reverse primers, adjusted with ultrapure water to a final volume of 10 µL. Thermal cycling conditions on the StepOne thermal cycler (Applied Biosystem, USA) comprised initial denaturation at 95 °C for 10 min, followed by 40 cycles of 95 °C for 15 s and 60 °C for 1 min, with a final step of 95 °C for 15 s, 60 °C for 1 min, and 95 °C for 15 s. Positive samples exhibited amplification with specific melting temperatures, while negative samples showed no specific amplification. As negative controls, DNA extracted from non-inoculated amoebas with purified viruses or samples was used, while DNA from amoebae infected with purified virus served as a positive control.

### Phylogenetic analyses

Some representative viruses of each group were sequenced. The samples containing purified virus underwent sequencing using the Illumina MiSeq system, employing a paired-end library and an Illumina DNA Prep kit (Illumina Inc., San Diego, CA, USA). Quality control of the obtained reads was conducted using the FastQC program, followed by read trimming with the Trimmomatic tool. Genome de novo assembly was performed using Spades 3.12 with default parameters [17].

Phylogenetic trees were constructed using IQtree software (version 1.6.12) employing the maximum-likelihood method, with 1,000 bootstrap replicates for branch support [18]. For tree construction, sequences of different genes were utilized. Data sets containing sequences for alignments were prepared using BLASTp against the NCBI non-redundant protein sequences (nr) database with an expected threshold of 10^− 3^ [19]. Alignments were performed using the Muscle software 3.8.1551 [20]. The best-fit substitution models were selected using the ModelFinder algorithm implemented in IQtree. Visualization and editing of phylogenetic trees were carried out using iTOL.

## Results

**Fig. 2:**
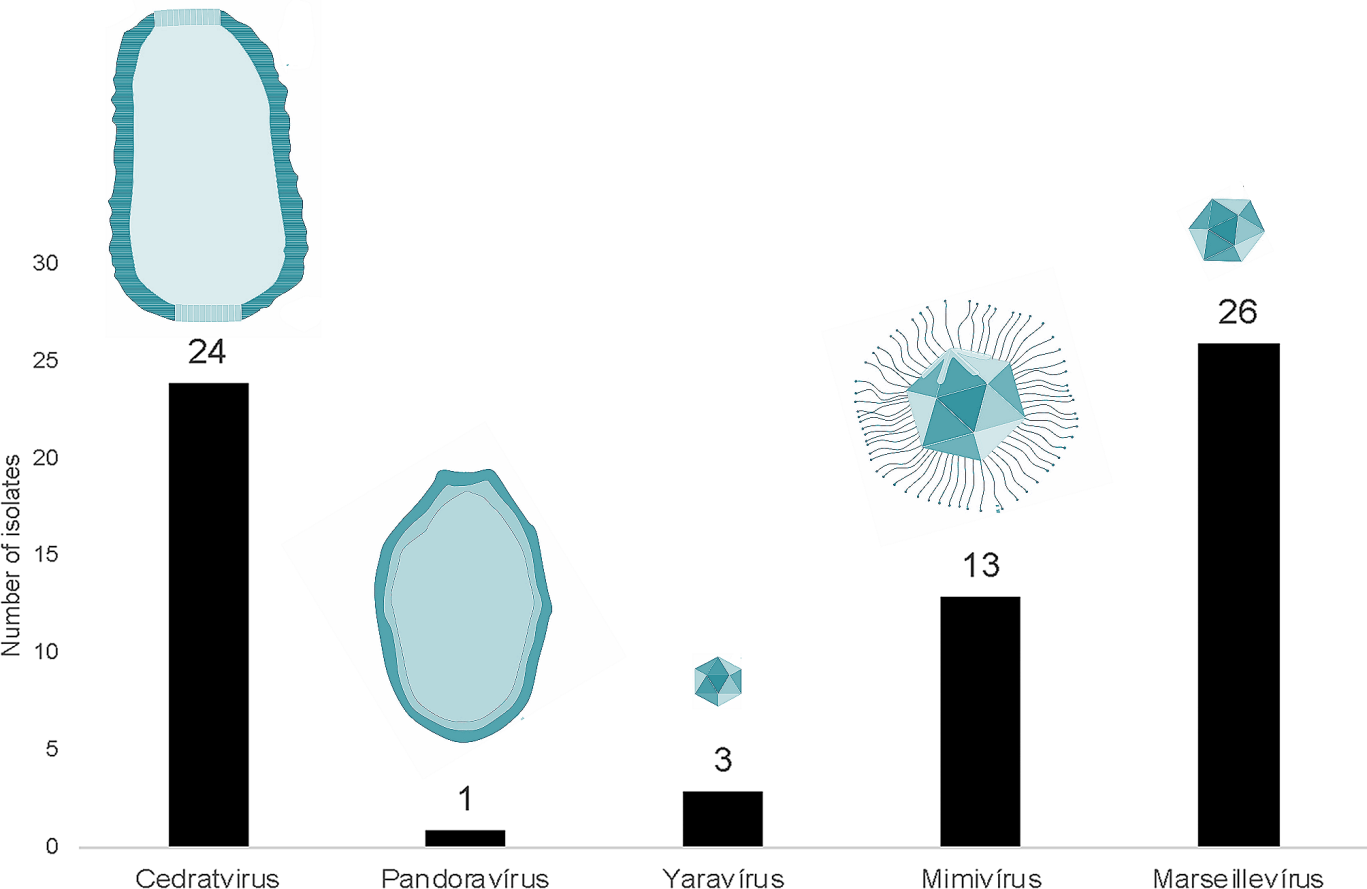
Number and variety of viruses isolated during this study. A total of 67 isolates were obtained from Brazilian biomes

In this prospective study, covering all Brazilian biomes, a total of 67 amoeba-associated viruses were isolated (Fig. 2). These viruses induced rounding and lysis as cytopathic effects in *Acanthamoeba*. Additionally, one of the isolates caused cell aggregation (Sup. Table 3). Only four isolates were obtained during the 1st passage, 11 isolates during the 2nd passage and the others (52) were detected during the 3rd passage of the prospecting procedures (Sup. Table 3). In addition, no isolates were obtained from the Amazon and Atlantic Ocean samples.

**Fig. 3:**
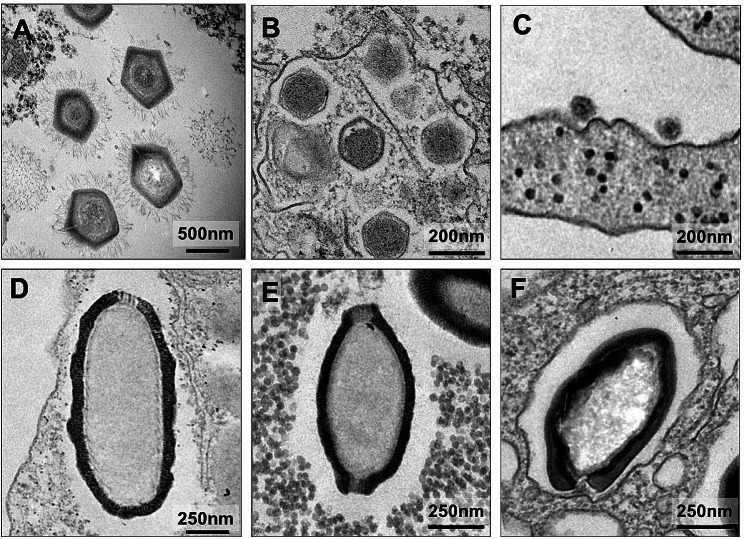
Transmission electron microscopy of isolated viral particles. (**A**) Mimivirus particles exhibiting its surface fibrils. (**B**) Marseillevirus particles with their typical icosahedral symmetry. (**C**) Yaravirus particles attached to the *Acanthamoeba castellanii* plasma membrane. (**D**) Cedratvirus particle with a single cork. (**E**) Cedratvirus particle with two corks. (**F**) Oval-shaped Pandoravirus particle, with its typical ostiole

**Fig. 4:**
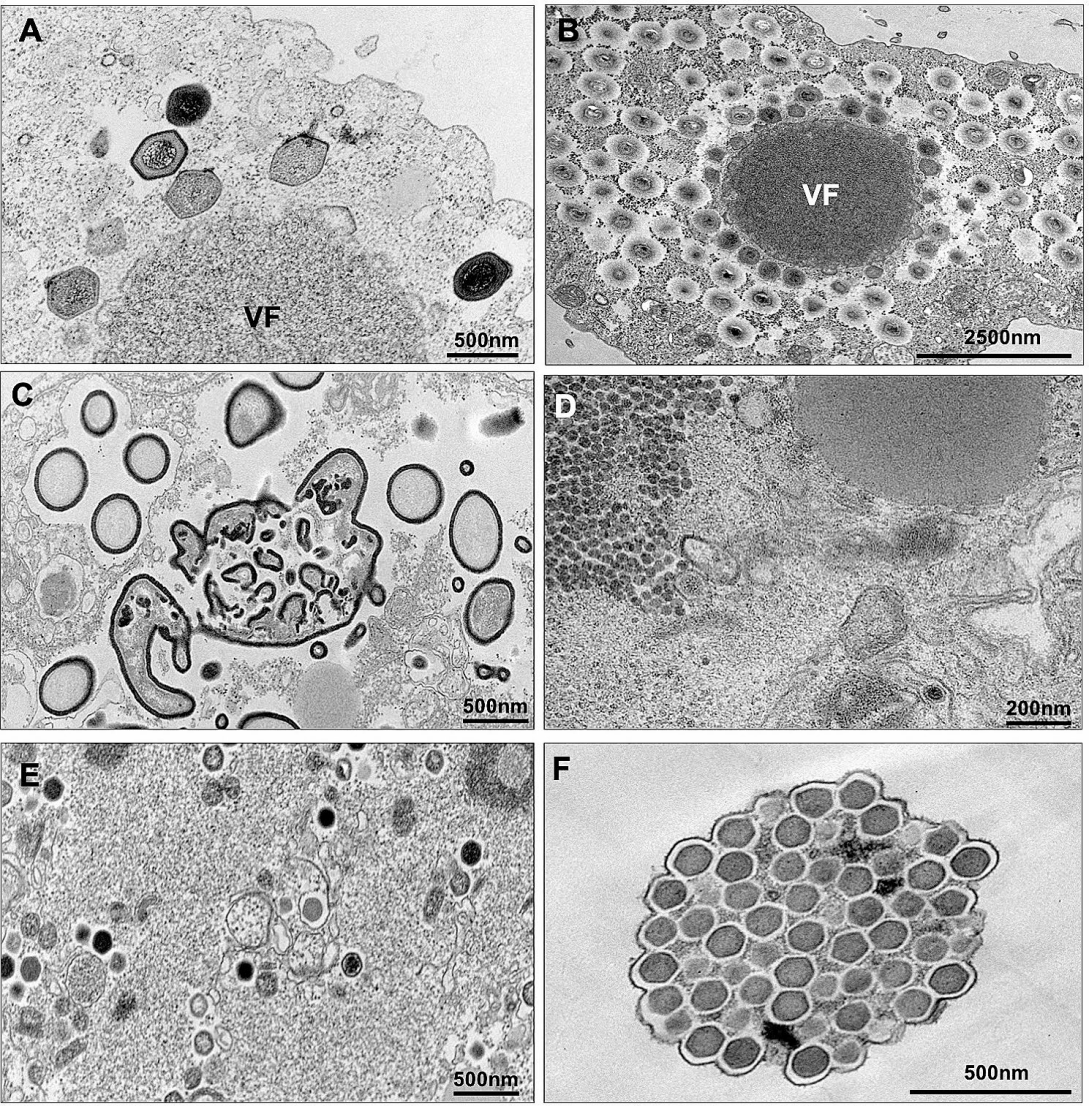
Transmission electron microscopy of ***Acanthamoeba castellanii*cells infected by different viral isolates. (A and B)** Mimivirus spherical electron-dense viral factory with particles under morphogenesis. VF – viral factory. (**C**) Cedratvirus viral factory. In the center, an amorphous structure that likely is related to the tegument morphogenesis. (**D**) Yaravirus viral factory. At top-left, genomic granular area. It is possible to visualize a particle at bottom-right (**E**) Marseillevirus electron-lucent viral factory. It is possible to visualize viral particles in different stages of morphogenesis. (**F**) A Marseillevirus giant vesicle containing dozens of viral particles

All isolates were first submitted to transmission electron microscopy (TEM) for identification (Figs. 2, 3 and 4). Here we will describe the types of isolated viruses and the criteria we used to identify them by TEM:

− 1 Pandoravirus isolate, with its typical ovoid shape particle and approximately 1 μm in length. It presents an apical ostiole, serving as an entry point for interactions with host cells. Within the host cell’s cytoplasm, an electron-lucent viral factory is discernible, indicative of active viral replication and assembly processes taking place (Figs. 2 and 3F).

− 13 mimiviruses, in which the viral structure exhibits pseudo-icosahedral symmetry, with a diameter measuring approximately 450 nm. Surrounding the capsid is a layer of fibrils, measuring approximately 125 nm in length. The capsid consists of multiple layers of protein. Enclosed within is an internal lipid membrane. Notably, the presence of the “stargate” feature. Within the host cell’s cytoplasm, an electron-dense viral factory is discernible, indicative of active viral replication and assembly processes (Figs. 2, 3A and 4A).

− 26 marseilleviruses, with icosahedral symmetry, these viral particles have a diameter of 180–250 nm. They may be observed individually or clustered together, inside vesicles. Within the host cell’s cytoplasm, an electron-luminous viral factory is evident, indicating active viral replication and assembly processes (Figs. 2, 3B and 4C and D).

− 24 pitho/cedrat-like viruses: The viral structure presents an ovoid shape, ranging from 600 nm to 1.5 μm in length. Its capsid is characterized by parallel vertical striations. Notably, one or two apical corks, also striated, can be observed. By electron microscopy, it is not possible to be sure if the isolate is a cedrat- or pithovirus, because particles with one or two corks have already been described for both groups of viruses. Within the host cell’s cytoplasm, an electron-luminous viral factory is noticeable, accompanied by the presence of electron-dense amorphous structures, indicative of active viral replication and assembly processes (Figs. 2, 3D and E and 4B).

− 3 yaraviruses: With icosahedral symmetry, the viral particles exhibit a diameter of approximately 80 nm. Within the host cell’s cytoplasm, the viral factory has two different areas: a granular, containing replicated genomic units; and an electron-lucent, containing empty capsids (Figs. 2, 3C and 4E).

Considering the identification of isolates by PCR, the viruses underwent reactions corresponding to all available targets, including Pandoravirus, mimivirus, Marseillevirus, cedratvirus, pithovirus, and Yaravirus. PCR results corresponded with TEM identification for all isolates, comprising 1 Pandoravirus, 13 mimiviruses, 26 marseilleviruses, 24 cedratviruses, and 3 yaraviruses. Therefore, the PCR indicated that all the isolates identified (Sup. Table 3) by TEM as pitho/cedrat-like viruses were, actually, cedratviruses.

Considering the increasing prevalence of giant virus prospective research in laboratories worldwide, we deem it pertinent and valuable to examine the relationships among variables such as substrates, biomes, and isolates. However, it is important to note that this analysis is descriptive in nature. As previously mentioned, the representativeness of explored substrates and biomes may not be equivalent due to logistical and financial constraints.

**Fig. 5:**
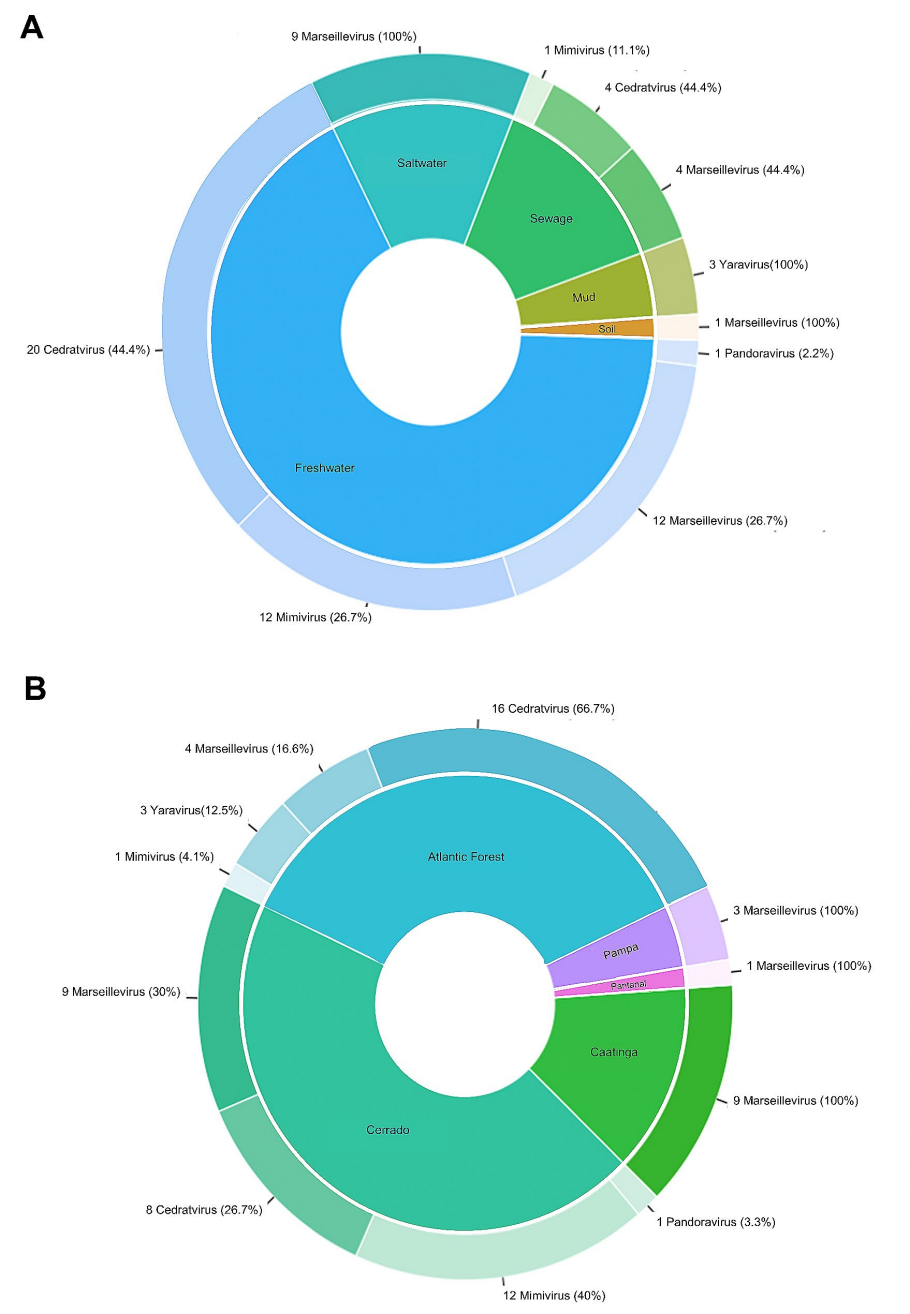
Isolates, samples and biomes. (**A**) Quantity and representativeness of each viral group isolated within each type of sample (substrate). The greatest variety of viral groups was obtained in freshwater. (**B**) Quantity and representativeness of each viral group isolated within each biome. The greatest variety of viral groups was obtained in Cerrado samples

New isolates were obtained from all types of samples tested (Fig. 5A). The largest quantity came from freshwater samples (45 isolates), which also exhibited the highest richness of isolated viral groups (at least 4 groups). From the less representative samples (soil and mud), only 1 Marseillevirus was isolated from soil samples, while 3 yaraviruses were obtained from mud samples. Saltwater and sewage samples yielded an equal number of isolates (9 isolates), but sewage samples showed greater richness of viral groups (3 groups) compared to saltwater samples (1 group).

The largest quantity of isolates was obtained from the Cerrado biome (30 new isolates), followed by the Atlantic Forest (24 isolates). However, the number of viral groups was the same for both (4 groups). From the Cerrado biome, isolates of Marseillevirus, mimivirus, Pandoravirus, and cedratviruses were obtained, while from the Atlantic Forest biome, isolates of mimivirus, Marseillevirus, cedratvirus, and Yaravirus were obtained (Fig. 5B).

**Fig. 6:**
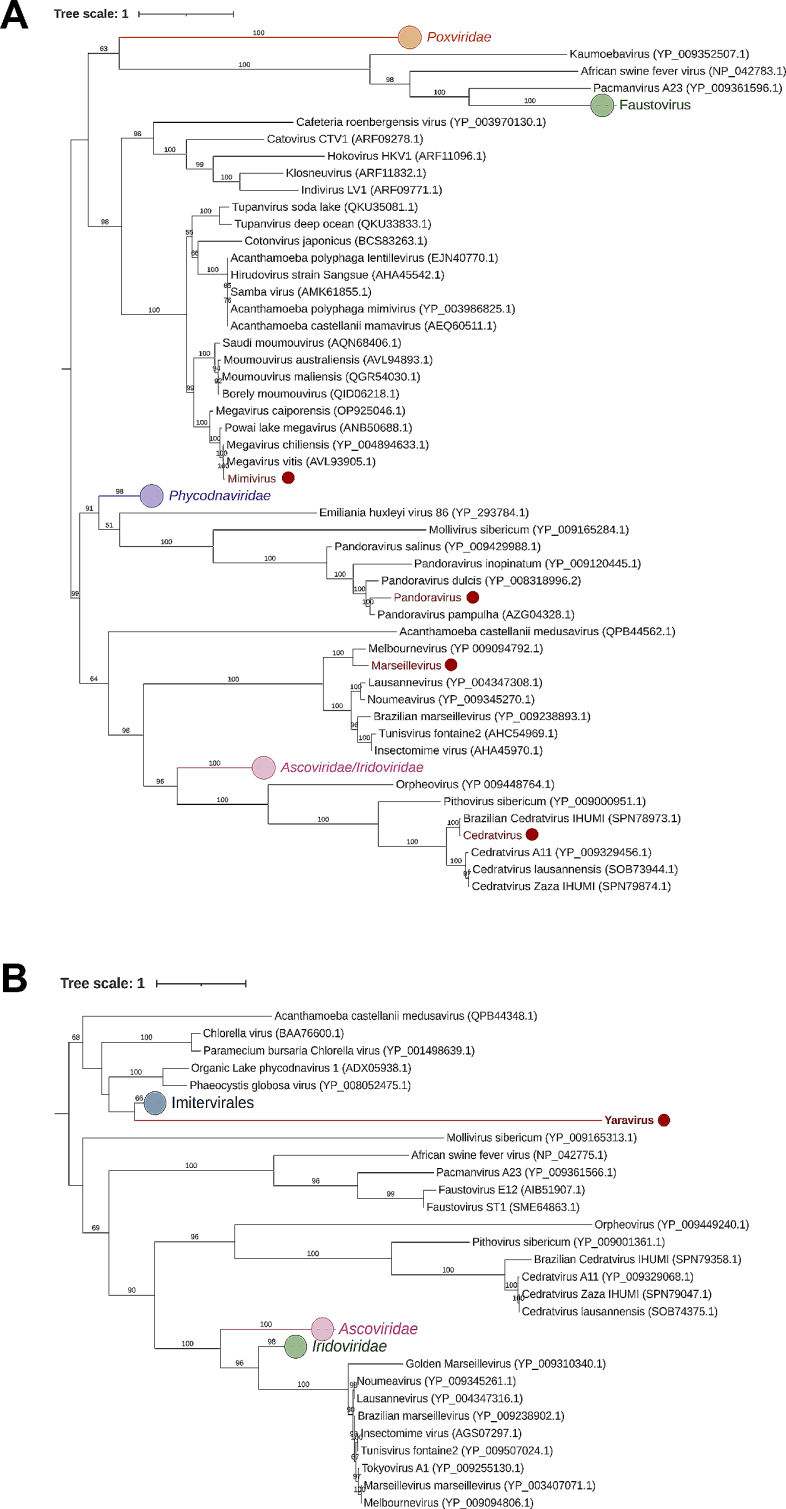
Maximum likelihood phylogenetic trees constructed with amino acid sequences from the DNA polymerase subunit B (mimivirus, Pandoravirus, Marseillevirus and cedratvirus) and the major capsid protein (Yaravirus). The new isolates described here are red highlighted. The scale bar indicates the genetic distance

In addition, representative viruses from each group were selected for genome sequencing to confirm their prior identification. For mimivirus, Pandoravirus, cedratvirus, and Marseillevirus, a phylogenetic tree was constructed using DNA polymerase, a common marker for giant virus phylogenetic analysis. In the case of Yaravirus, the major capsid protein (MCP) gene was utilized for tree construction. The results indicate that the previous identification of the isolates through TEM and PCR aligns with phylogenetic analysis, confirming the identification of the viruses (Fig. 6).

## Discussion

The remarkable biodiversity of organisms in Brazilian natural and urban environments pose questions and supports virology studies employing a prospecting approach as employed in this study. Consequently, the ongoing isolation and characterization of giant viruses may contribute to the understanding of their ecological roles, evolutionary dynamics, and potential impact on host populations and ecosystems. In this sense, obtaining several environmental samples from different biomes, collected over a span of 11 years yielded a total of 67 new isolates belonging to at least five distinct viral groups, including Pandoravirus, mimivirus, Marseillevirus, cedratvirus, and Yaravirus. Over time, other prospecting studies on amoebal viruses were also performed in Brazilian biomes. Dornas et al. (2015) and Andrade et al. (2019) described the isolation of different types of *Acanthamoeba*-infecting giant viruses. However, in contrast to the present study, mimivirus was previously the most frequently isolated virus.

Information on the distribution patterns of isolated viruses across different biomes and sample types may be useful for future prospecting studies. Despite logistical and financial constraints, our study successfully captured the diversity of giant virus diversity present in Brazilian environments in a span of 11 years of research. However, it is important to acknowledge the limitations of our sampling approach, particularly regarding the representativeness of explored substrates and biomes. Future studies should aim to address these limitations and further explore the ecological factors driving the distribution and diversity of giant viruses in Brazil and beyond.

Importantly, to an extended overview of such remarkable viral entities, our results suggest that morphological identification by TEM was effective in distinguishing between different viral groups. However, distinguishing between cedratviruses and pithoviruses proved challenging due to their strikingly similar characteristics. Therefore, additional PCR analysis or genome sequencing proved necessary to resolve identification ambiguities for these viruses. Last, phylogenetic analysis of representative viruses from each group further validated their taxonomic classification and evolutionary relationships. In conclusion, by combining such approaches we have expanded our understanding of the distribution of the giant viruses in Brazilian biomes. It is important to mention that some of the viral isolates described here have already undergone genomic and biological characterization [15, 21, 22]. This underscores the significance of prospecting studies as the initial step in knowledge construction.

### Electronic supplementary material

Below is the link to the electronic supplementary material.

Supplementary Material 1

Supplementary Material 2

Supplementary Material 3

## Data Availability

Viral sequences are available at Genbank under the accession numbers MT293574, OR343515, OR991738, MK131393.

## Declarations

### Sequencing data

Viral sequences are available at Genbank under the accession numbers MT293574, OR343515, OR991738, MK131393.
