# Supplementary material for: A long-term prospecting study on giant viruses in terrestrial and marine Brazilian biomes
Source: Virol J. 2024 Jun 10;21:135. doi: 10.1186/s12985-024-02404-z (PMC11165748; doi:10.1186/s12985-024-02404-z)
Supplement: Supplementary file 2 — Supplementary Material 2 [file 12985_2024_2404_MOESM2_ESM.docx]

| **Supplementary table 2:** primers used in this study | |  |  |  |
| --- | --- | --- | --- | --- |
| **Virus** | **Target** | **Primer F** | **Primer R** | **Source** |
| Mimivirus | major capsid protein | 5’-ACTTTATTATCATTATCAGCGAATA-3’ | 5’-GCTCTTAACCCTGAAGAACA-3’ | doi: 10.1186/s12985-018-0930-x |
| Marseillevirus | major capsid protein | 5’-CTTTTGCACCTGCTTCATGA-3’ | 5’-GCGGTAACCCTCCCACTTAT-3’ | doi: 10.1186/s12985-018-0930-x |
| Pandoravirus | Main protein of the capsid | 5’-GGATGGCTCGACGTCTCTT-3’ | 5’-CCTYGGTRAGCAMAGGCAAC-3’ | doi: 10.1186/s12985-018-0930-x |
| Cedratvirus | Main protein of the capsid | 5′- AGAGTATGCTCGCAACCACC-3’ | 5’-CACGTTAAGGCCGGGGTAAT −3’ | doi: 10.1186/s12985-018-0930-x |
| Yaravirus | Gene 69 | 5′- TGCAGCAAGTCGGTCAAGAT-3′ | 5′-AACTTCCACATGCGAAACGC-3' | doi: 10.1073/pnas.2001637117 |
| Pithovirus | DNA/RNA helicase | 5' - GTGGTCCAAAACTGGAAGAACTA-3' | 5'-GCGTCAAGCTCAACATCAAGTTC-3' | doi: 10.3390/v15020564 |
